# Vitamin-D receptor (VDR) gene polymorphisms (Taq-I & Apa-I) in Syrian healthy population

**DOI:** 10.1016/j.mgene.2014.08.005

**Published:** 2014-09-14

**Authors:** Shaden HADDAD

**Affiliations:** Biochemistry and Microbiology Department, Faculty of Pharmacy, Damascus University, Syria

**Keywords:** VDR gene polymorphism, PCR-RFLP, Syria

## Abstract

The vitamin D endocrine system regulates bone metabolism and calcium homeostasis as well as cellular proliferation and differentiation. Vitamin D receptor (VDR) mediates Vit-D activity, thus VDR gene polymorphisms may correlate with different diseases. This study aimed to determine the distribution of VDR gene (Taq-I and Apa-I) polymorphisms using a RFLP in unrelated normal healthy individuals of Syrian population. Allelic frequencies were 65% vs 35% and 66% vs 34% for T vs t and A vs a alleles, respectively. Genotype distribution was 36%, 58% and 6% for TT, Tt and tt and 42%, 47% and 10% for AA, Aa and aa, respectively. These results demonstrate that the frequency and distribution of the VDR polymorphisms in Syrian population are different from other populations worldwide.

## Introduction

The vitamin D receptor (VDR) belongs to the steroid hormone receptor family and mediates the effects of 1,25-dihydroxyvitamin D (1,25(OH)2D) on gene expression (Walters, 1992). The VDR gene is distributed on chromosome 12q12-q14. Eight exons (2–9) and six alternatively spliced regions (1a–1f) are located in functionally relevant areas, including the promoter region. Several polymorphisms have been identified in the VDR gene (Uitterlinden et al., 2004). The 3′ untranslated region of the VDR gene includes Apa-I (Faraco et al., 1989), Bsm-I (Morrison et al., 1992) and Taq-I (Morrison et al., 1994). The potential effects of VDR polymorphisms on disease susceptibility have been investigated (Zmuda et al., 2000). These effects could be altered by the differences in race and diet (Valdivielso and Fernandez, 2006). Most of the polymorphisms used have an unknown functional effect. This effect could be due to a linkage to truly functional polymorphisms elsewhere in the same or a nearby gene. In the case of the differences in RNA, or changes in a totally different gene, the VDR polymorphisms would act as a marker of truly functional polymorphisms elsewhere. Thus, the association of a certain polymorphism with a phenotype does not necessarily mean that the polymorphism is causing it. The association of alleles of different polymorphisms with each other within a population is called linkage disequilibrium (LD) (Wall and Pritchard, 2003). Blocks of alleles that are present together with high levels of LD are called haplotype. Taq-I and Apa-I variants are the most studied VDR polymorphisms (Morita et al., 2004). In Syria, studies of the association between VDR gene polymorphisms and diseases such as postmenopausal osteoporosis and asthma are still in progress. Another study of the association of VDR polymorphism Bsm-I with type 1 diabetes mellitus has been completed and published (Al-Moubarak Samah, 2013). This study aims to investigate the distribution of VDR (Taq-I and Apa-I) gene polymorphism in unrelated healthy individuals in Syria.

## Materials and methods

Blood samples were collected on EDTA from 78 unrelated normal individuals (Male 37 & Female 41) from Syrian population. DNA was extracted from blood using commercial kit (Fermentas kit). The restriction fragment length polymorphisms (RFLP) of the VDR gene were determined by polymerase chain reaction (PCR) amplification and digestion of the products with ApaI and TaqI. A region of 2000 bp carrying the polymorphic restriction sites of Apa-I and Taq-I was amplified by PCR. The specific primers for the VDR were forward primer (5′CAACCAAGACTACAAGTACCGCGTCAGTGA-3′) in intron 8 (Morrison et al., 1994) and a reverse primer (5′CACTTCGAGCACAAGGGGCGTTAGC-3′) in exon 9 (Sainz et al., 1997).

The total volume of amplified genomic DNA was 50 μl containing 20–100 ng DNA, 25 μl of master mix PCR (Fermentas) and 15 pmol of each primer (VBC-biotech). PCR amplification was carried out in a DNA Thermal Cycler (Biocycler TC-S). PCR conditions were 3 min for initial denaturation at 94.5 °C; 35 cycles at 94.5 °C for 1 min for denaturation, 1 min at 61 °C for annealing and 2 min at 72 °C for extension, followed by 7 min at 72 °C for final extension (Gursoy et al., 2008). The PCR amplified product (2000 bp) was digested for 5 h at 65 °C with Apa-I (MBI Fermentas, Vilnius, Lithuania), and digestion for 3 h at 65 °C Taq-I (MBI Fermentas, Vilnius, Lithuania). The digest products were resolved on a 1.5% agarose gel containing 0.5 μg/ml ethidium bromide using a gel electrophoresis system (Pequlab, Germany) at 100 V for 20–30 min. The gel was visualized under UV light using a transilluminator system (Cleaver Scientific Ltd., UK).

The genotyping and digest product lengths were AA 2000 bp; Aa 2000 bp, 1700 bp and 300 bp; aa 1700 bp and 300 bp; TT 2000 bp; Tt 2000 bp, 1800 bp and 200 bp; and tt 1800 bp and 200 bp. Using the restriction endonucleases Apa-I and Taq-I, the genotypes defined were characterized as A, T (indicating the absence of the restriction site) or a, t (indicating the presence of the restriction site), respectively (Riggs et al., 1995). Genotype frequencies of the VDR gene polymorphism in Syrian population were determined according to Hardy–Weinberg equilibrium and data were analyzed using the computer software SPSS (version 19) and Prism 4.

## Results

The frequencies (%) of alleles and genotypes of the Apa-I and Taq-I loci in Syrian population are presented in Table 1.

The allelic frequency of ‘T’ vs ‘t’ and ‘A’ vs ‘a’ were 65 vs 35% and 66 vs 34% respectively in Syrian population. Comparison of frequency distribution of different genotypes and alleles of VDR gene was done between Syrian and different populations that mentioned in North Indian study (Bid et al., 2005), Turkey (Gunes et al., 2008), Iran (Nosratabadi et al., 2010) and Jordan (Karasneh et al., 2013) using χ^2^ tests. In this study, no differences in the distribution of genotypes and alleles of Taq-I were found between Syrian individuals and each of the populations of Jordan, Sweden, Black Pennsylvania and Australia as shown in Table 2.

In this table, the data were adapted basically from the North Indian study (Bid et al., 2005) and studies from Jordan (+) (Karasneh et al., 2013), Turkey (+) (Gunes et al., 2008) and Iran (+) (Nosratabadi et al., 2010). Whereas the distribution of genotypes and alleles of Apa-I was similar in Syrians compared to populations of Jordan, North India, Turkey, France, Greece and Black Pennsylvania as shown in Table 3. In this table, the data were adapted basically from the North Indian study (Bid et al., 2005) and studies from Jordan (+) (Karasneh et al., 2013), Turkey (+) (Gunes et al., 2008) and Iran (+) (Nosratabadi et al., 2010).

## Discussion

The importance of VDR polymorphism study in population arises from the differences between genotypes and alleles according to the ethnicity. This requires comparison of genotypes and allele frequency between healthy individuals and patients in each population and then comparing the genotype and allele frequency with other different populations. In this study, the genotypes and allele frequency of VDR (Taq-I and Apa-I) were reported in Syrian population and compared with those reported in different studies worldwide as shown previously. There are differences among different populations as presented in Table 2, Table 3. Syrian population was compared to all other different populations that mentioned in the North India study (Bid et al., 2005) in addition to populations of Iran, Turkey and Jordan (Karasneh et al., 2013), which are Asian countries.

The results of both polymorphisms (Taq-I and Apa-I) of Syrian population were consistent with the results of populations of Jordan (Karasneh et al., 2013), and Black Pennsylvania (Zmuda et al., 1997). On the contrary, there was significant difference in genotype and allele distribution (Taq-I and Apa-I) between Syrian population and Asian populations: Japan (Tokita et al., 1996), China (Kung et al., 1998), Thailand (Ongphiphadhanakul et al., 1997), and Iran (Nosratabadi et al., 2010). This present study demonstrated the effect of ethnicity on VDR gene variations. Many studies mentioned the role of VDR gene polymorphisms in disease susceptibility especially diseases related to calcium metabolism such as osteoporosis. The Japanese study presented that the people with aa genotype have more lumbar spine BMD than people with AA genotype (Tokita et al., 1996). The same result was found in a Mexican study, wherein descent girls with aa genotype had 2 to 3% higher femoral bone density and 8 to 10% higher vertebral bone density than girls with AA (Sainz et al., 1997). Whereas there was lack of a heritability pattern between the AA genotype and low BMD in Greece (Fountas et al., 1999).

## Conclusion

This study demonstrates the genotypes and allele frequency of VDR gene polymorphism (Apa-I and Taq-I) in Syrian healthy individuals. These results show the impact of ethnicity on VDR polymorphism distribution in healthy populations. This will support our understanding of the relationship between the VDR polymorphisms and diseases in Syrian population. Also, it will explain the effect of ethnicity on disease susceptibility when the patients of different populations will be compared to Syrian population.

## Figures and Tables

**Table 1:** Genotype and allele frequency of VDR polymorphisms (Taq-I and Apa-I) in Syria.

	Genotypes n (%)			Alleles %	
N = 78	TT 28 (36)	Tt 46 (58)	tt 5 (6)	T 65	t 35
N = 78	AA 33 (42)	Aa 37 (47)	aa 8 (10)	A 66	a 34

**Table 2:** Comparison of genotypes and allele frequency of VDR gene polymorphism (Taq-I) between Syrian and different populations.

Country	No.	Genotype (%)				Alleles (%)		
TT	Tt	tt	P	T	t	P
Syria	78	36	58	6	Ref	65	35	Ref

*Asia*								
Jordan +	126	32.5	47.6	19.8	NS	56.4	43.6	NS
Japan	488	77	22	1	***	88	12	***
China	144	90	10	0	***	95	5	***
Thailand	84	83	17	0	***	92	8	***
North India	346	49	40	11	*	66	34	NS
Turkey +	102	47	35.2	6.9	*	70	30	NS
Iran +	100	18	35.5	47	***	35.5	64.5	***

*Europe*								
France	189	33	49	18	*	57	43	NS
Austria	163	12	49	39	***	36	64	***
Sweden	100	34	54	12	NS	61	39	NS
Greece	53	38	41	21	**	59	41	NS

*Americas United States*								
White, Minnesota	130	41	44	15	*	63	37	NS
Black Pennsylvania	101	32	53	15	NS	58	42	NS
Mexican, California	101	51	40	9	*	71	29	NS

*South Pacific*								
Australia	518	36	48	16	NS	60	40	NS

* = p < 0.05, ** = p < 0.01, *** = p < 0.001, NS = not significant.

**Table 3:** Comparison of genotypes and allele frequency of VDR gene polymorphism (Apa-I) between Syrian and different populations.

Country	No.	Genotype (%)				Alleles (%)		
AA	Aa	aa	P	A	a	P
Syria	78	42	47	10	Ref	66	34	Ref

*Asia*								
Jordan +	126	41.3	44.4	14.3	NS	63.5	36.5	NS
Japan	488	9	48	43	***	33	67	***
China	144	10	36	54	***	29	71	***
Thailand	84	11	50	39	***	36	64	***
North India	150	36	44	20	NS	58	42	NS
Turkey +	102	39.2	42.2	18.6	NS	60	40	NS
Iran +	100	17	56	27	***	45	55	**

*Europe*								
France	189	30	50	20	NS	54	46	NS
Austria	163	29	45	26	**	52	48	NS
Sweden	100	27	52	21	*	53	47	NS
Greece	53	36	43	21	NS	58	42	NS

*Americas United States*								
White, Minnesota	128	30	46	24	*	53	47	NS
Black Pennsylvania	101	44	46	10	NS	67	33	NS
Mexican, California	100	21	55	24	**	48	52	*

*South Pacific*								
Australia	518	26	51	23	*	51	49	*

* = p < 0.05, ** = p < 0.01, *** = p < 0.001, NS = not significant.
